# Canada Medical Journal

**Published:** 1852-05

**Authors:** 


*Canada Medical Journal.*

The *Canada Medical Journal*, edited by Dr. R. S. Macdonnel and Dr. A. H. David, has been received. This journal has taken the place of the "*British American Journal of Medical and Physical Sciences.*" The number received has several of its articles printed in French, which fact, we presume, will render it very acceptable to many practitioners in the province, and others who are acquiring a knowledge of that language. We welcome it to our exchanges. J

